# Preliminary *in vitro* hemolysis evaluation of MR-conditional blood pumps

**DOI:** 10.3389/fmedt.2025.1671938

**Published:** 2025-10-23

**Authors:** Dominik T. Schulte, Carolina Pietsch, Natalija Topalovic, Michael Hofmann, Martin O. Schmiady, Miriam Weisskopf, Marianne Schmid Daners

**Affiliations:** ^1^ Institute for Dynamic Systems and Control, ETH Zurich, Zurich, Switzerland; ^2^ Center for Surgical Research, University Hospital Zurich, University of Zurich, Zurich, Switzerland; ^3^ Center for Cardiac Surgery, University Hospital Zurich, University of Zurich, Zurich, Switzerland

**Keywords:** MR-conditional, blood pump, extracorporeal membrane oxygenation, hemolysis, *in vitro* testing, cardiopulmonary bypass

## Abstract

**Purpose:**

Magnetic resonance imaging (MRI) during cardiopulmonary bypass is hindered by the incompatibility of conventional heart-lung machines, which contain metallic components that interfere with the MRI environment. This study evaluates the hemolytic performance of three MR-conditional blood pump prototypes—roller, non-occlusive roller, and centrifugal—designed for use during neonatal surgery.

**Materials and methods:**

Each pump was tested using acid-citrate dextrose-stabilized bovine blood at a neonatal-relevant flow rate of 1 L/min. Due to limitations of the setup, a low pressure head of 10 mmHg was applied uniformly across experiments. Hemolysis was assessed using normalized index of hemolysis, and a linear mixed-effects model was applied to account for experimental variability.

**Results:**

The roller pump showed the lowest hemolysis (1.84 ± 1.90 mg/100 L). The centrifugal pump showed the highest (8.43 ± 1.63 mg/100 L), alongside mechanical leakage. Random effects (SD = 2.07) indicated moderate inter-experimental variability.

**Conclusion:**

While all prototypes performed comparably to standard references under controlled conditions, further testing at physiological pressure levels and stricter adherence to ASTM F1841 is necessary for broader validation.

## 1 Introduction

Cardiopulmonary bypass (CPB) is a well-established technique in adult cardiac surgery and is used in roughly 2,000 procedures worldwide each day (1, 2). However, its application in neonates — particularly those with congenital heart defects — has drawn increasing research attention. Studies examining the effects of heart-lung machines (HLMs) in this population have reported mixed outcomes, with some cases showing white matter brain injuries emerging soon after birth (3). In most cases, undergoing cardiac surgery with CPB was associated with a 20%–50% rise in the occurrence of white matter brain injuries (4–6), although this is currently subject of debate (7).

Magnetic resonance imaging (MRI) is the primary tool for detecting white matter brain injuries and is usually performed before and after cardiac surgery. Using it during surgery, however, would allow direct investigation of cerebral perfusion dynamics in the infant brain. However, real-time imaging during surgery remains unfeasible due to the presence of ferromagnetic components in conventional HLMs, which are unsafe in the MRI environment. This limitation is especially critical in neonates, whose low total blood volume of approximately 85 mL/kg requires that the HLM be placed particularly close to the patient, such that the tubing can be kept as short as possible (8, 9). A strategy by Schuster et al. (10) involved using long polycarbonate drive shafts to transmit mechanical power from outside the MR suite; however, this setup is impractical due to its large spatial footprint. An alternative solution is to utilize an MR-conditional actuation system, such that the full HLM can operate safely within the MRI environment — a solution that could also be extended to extracorporeal membrane oxygenation (ECMO) systems. In ECMO applications, such MR-conditional blood pumps would enable patients to undergo advanced imaging without interruption of life-sustaining therapy.

Previous research has demonstrated the feasibility and hydraulic performance of three MR-conditional pump designs, namely a roller pump, a non-occlusive roller pump and a centrifugal pump (11, 12). To safely operate a heart-lung machine (HLM) inside an MR scanner, apart from demonstrating MR compatibility and sufficient flow generation, the potential for blood damage caused by the components of a CPB system needs to be investigated as well. Mechanical stress on red blood cells can lead to hemolysis, in which the cells rupture and release their contents into the plasma (13). This study aims to fill this research gap by comparing the hemolytic effects of the three MR-conditional pumps, namely the roller pump, non-occlusive roller pump and centrifugal pump, to identify the most suitable option for future *in vivo* studies investigating possible causes for white matter injuries during cardiac surgeries in neonates.

## 2 Materials and methods

**Figure 1:**
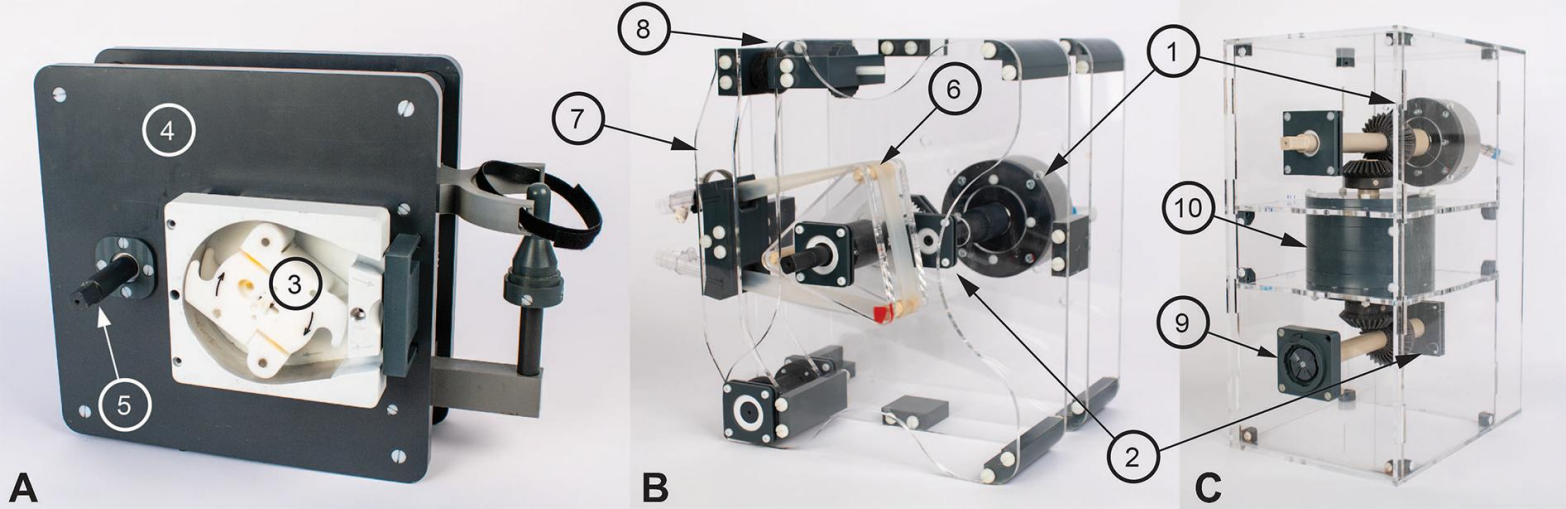
The three developed magnet resonance (MR)-conditional pumps. The PM0450 air motor (1) and the ME 22 LD encoder (2) were used in all three pumps (cannot be seen in A). **(A)** shows the roller pump with the SPQ 225 pump head (3), the polyvinyl chloride pump body (4), and the emergency crank (5). **(B)** shows the non-occlusive roller pump with its three polymer rods (6), and the tensioning arm (7) that can be adjusted with a threaded rod (8). **(C)** shows the centrifugal pump with the mechanical transmission (9) to the DeltaStream DP2 pump head and the two-stage planetary gears (10) (14).

All three above-mentioned prototypes (Figure 1) are actuated using a PM0450 air motor (PTM Mechatronics GmbH and Bibus AG, Fehraltorf, CH). The roller pump features a modified SPQ 225 pump head (Möller Medical GmbH, Fulda, DE), in which the original springs were replaced with closed-cell polyurethane foam (Sylodyn NC, Getzner Werkstoffe GmbH, Bürs, AT). The non-occlusive roller pump is constructed entirely from milled polyvinyl chloride (PVC) and acrylic plates, using standard 1/2’’ silicone tubing (HMT Medizintechnik GmbH, Maisach, DE) stretched over three polymer rods. The centrifugal pump is based on a modified DeltaStream DP2 model (MEDOS Medizintechnik AG, Heilbronn, DE), incorporating a polyetheretherketone (PEEK) shaft, ceramic ball bearings, and a PVC part for non-magnetic transmission. Its actuation is driven via a custom two-stage planetary gearbox using thermoplastic gears (Stagnoli TG s.r.l., Lonato, IT). Replacing the magnetic coupling on the DP2 caused the back of the pump to remain open, causing slow leakage through the ball bearings and mechanical coupling.

The two roller pumps and the centrifugal pump were compared to two off-the-shelf reference pumps: a LivaNova Sorin Stockert S5 (SOMA TECH INTL, Bloomfield CT, US) and an electrically actuated DeltaStream DP2, respectively. Since no commercial version of the non-occlusive roller pump is available, its hemolysis performance was compared with the conventional roller pump, given their shared underlying functional principle.

### 2.1 Hemolysis evaluation

The ASTM F1841 (15) is a core guideline providing detailed instructions on conducting a hemolysis study, covering setup, pump conditions, blood choice and evaluation. While this standard was used as a general reference for the preliminary study, several practical constraints required deviations from its protocol. Details of the deviations are described in the following paragraphs. Despite these differences, all prototypes were tested in parallel under identical conditions, using blood from the same donor animal for each individual experiment (16). Bovine blood (S0025000, Bioswisstec AG, Schaffhausen, CH) was used for the hemolysis experiments. It was anticoagulated with acid-citrate dextrose before shipping and was specified to have a standardized hematocrit of 32% ± 3%.

**Figure 2:**
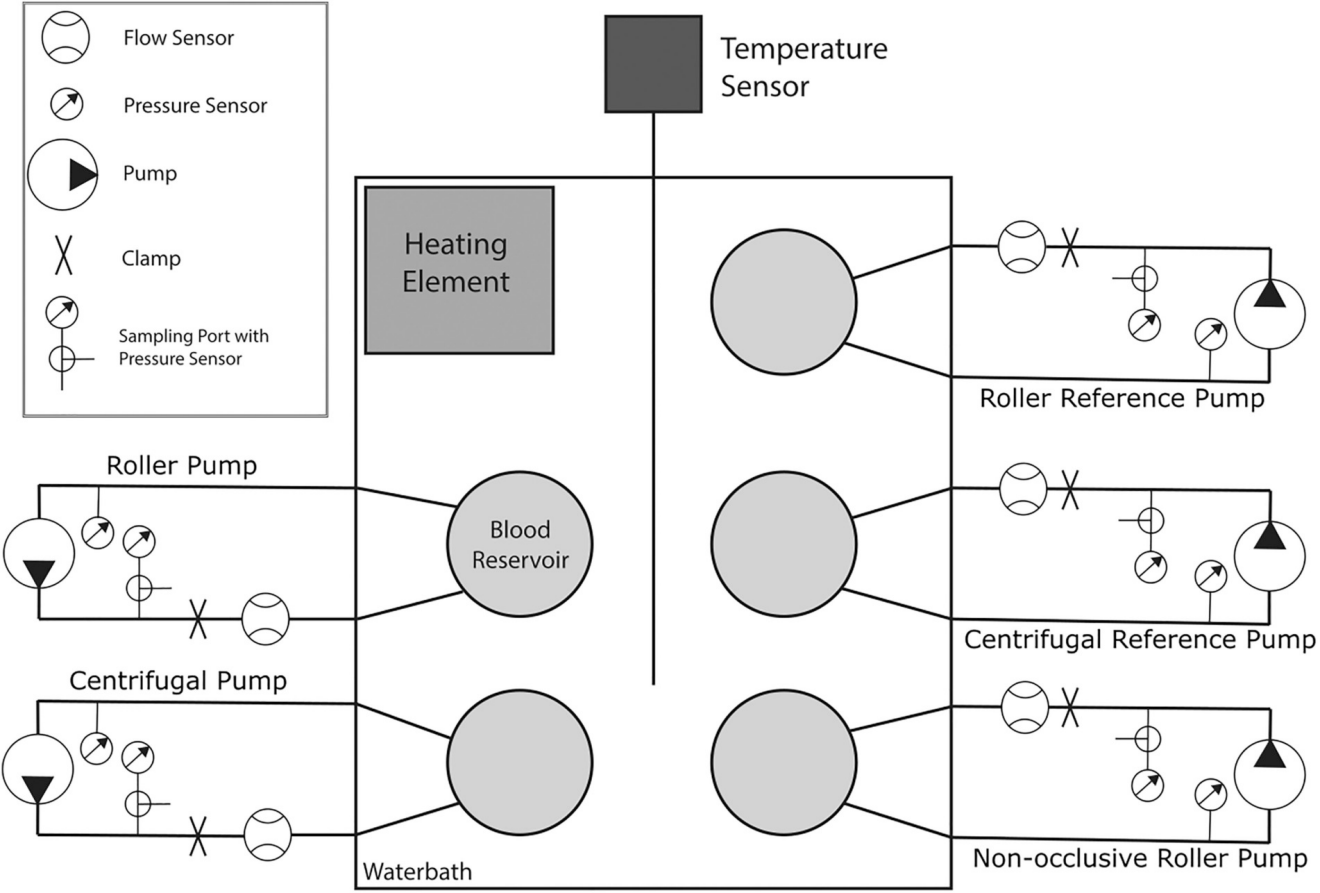
Schematic representation of the hemolysis setup, consisting of five separate systems. Each system consists of a flow sensor, a clamp for adjusting the pressure head, a pressure sensor upstream and downstream of the pump for measuring the pressure head, a blood reservoir, and a sampling port with a three-way connector at the second pressure sensor. The reservoirs are placed in a bath containing saline solution, the temperature of which is measured by a temperature sensor and is maintained at a constant temperature of 37.5 ± 1 °C by the heating element.

Each pump was connected to a circuit consisting of a flow sensor (Sonoflow CO.55/190, Sonotec, Halle, DE), two pressure sensors (Meritrans DTX Plus, Meritmedical, South Jordan UT, US), a clamp (to set the pressure head), and an open reservoir (Figure 2). These reservoirs were placed without a lid in an also open heating bath with water for the first experiments, which was changed to 0.9% saline solution after the fourth experiment to prevent hemolysis in case of accidental fluid ingress into the blood circuit. Since the reservoirs had no lid, gas exchange with the surrounding air was possible, whereas the rigid walls of the reservoirs and the tubing restricted exchange. The bath was heated with a Corio CD (Julabo GmbH, Seelbach, DE) to 37.5 ± 1 °C and the temperature measured with a Luxtron 812 Fiber Optic Thermometer (Advanced Energy, Denver CO, US). Each circuit was first flushed with saline solution to ensure cleanliness, then primed with 420 mL of blood for the initial run. In subsequent runs, priming volumes were adjusted to account for issues such as mechanical leakage in the centrifugal pump. All priming volumes were recorded for consistency.

After priming, the pumps were slowly turned on one after the other and set to the target flow speed. The ASTM guideline (12) defines a pump flow of 5 ± 0.25 L/min, however, according to ELSO guidelines (17), recommended flow rates are 100 mL/kg/min for neonates and 80 mL/kg/min for pediatric patients. To be closer to a clinically relevant setpoint a flow setpoint of 1 L/min (simulating a pediatric patient of around 12.5 kg of body weight) was used in our study.

The ASTM guideline further recommends a pressure head of 500 ± 15 mmHg for CPB application; however, the centrifugal pumps were unable to reach such a clinically relevant pressure head, achieving only around 10 mmHg. To maintain consistency across all tests, a pressure head of 10 mmHg was applied to each pump. Although this configuration may underestimate hemolytic stress compared to clinical conditions, it allowed for a controlled comparison of the three prototypes and their respective references under identical flow conditions.

Each experiment ran for six hours and was repeated seven times. Blood samples of 1.5 mL each were taken every 30 min. Each blood sample was placed in a centrifuge for 10 min at 3,000 rcf to separate the red blood cells from the plasma. For each sample, three sub-samples were prepared by mixing 100 μL of plasma and 1,000 μL of $0.1\text{\%}\;Na_{2}CO_{3}$ solution. Each of these sub-samples was placed in the photometer (4040 photometer, Robert Riele GmbH & Co. KG, Berlin, DE), which was measured at three different wavelengths of 380 nm, 415 nm, and 450 nm to determine the amount of free hemoglobin (fHb) found in the blood plasma, according to the method described by Harboe (18).

The fHb value was averaged over the three sub-samples. These averages were then used to calculate the normalized index of hemolysis (N.I.H.) (Equation 1) according to the ASTM F1841 (15).

(1) $$\begin{array}{c} N.I.H.\;\left(\frac{g}{100L}\right)\;=\Delta fHb\;*\;V\;*\;\frac{100-Ht}{100}\;*\;\frac{100}{Q\;*\;T}\; \end{array}$$

in grams of fHb produced per 100 L of blood pumped, with ΔfHb being the increase in plasma free hemoglobin concentration over the sampling interval (g/L), V the volume of the circuit (L), Ht the hematocrit (%), *Q* the flow rate (L/min), and T the sampling time interval (min). For each pump, the measured fHb and the resulting indices were reported as a mean with the corresponding standard deviation for each experiment.

Outliers were removed prior to calculations according to the following criteria. Artefacts in fHb measurements were detected by their characteristic pattern: a single erroneous value produced two consecutive index values that were extreme and of opposite sign. Such paired values are physiologically implausible and were excluded from further analysis. Water ingress into the circuits was detected due to a rapid discoloration of the blood in the circuit. At the same time a very pronounced and steep increase in fHb could be observed from the measurement. After changing the solution to saline, this was not observed any longer.

Additionally, another sample was taken every 60 min and analyzed using a hematology analyzer (Vetscan HM5, Zoetis, Parsippany-Troy Hills NJ, US) to measure hematocrit and mean corpuscular volume (MCV) value and an EPOC blood gas analyzer (Siemens Healthineers, Erlangen, DE) to assess pH value.

### 2.2 Data assessment and statistical analyses

While the previous section focused on the direct interpretation of raw data, the following section applies advanced statistical tools to further analyze the measurements and derive deeper insights. The differences in the mean values between the hemolysis indices were analyzed by applying a linear mixed-effects model, as well as the corresponding confidence intervals. A linear mixed-effects model is deemed useful when measurements are taken repeatedly on the same subjects, especially when data within one group are more similar to each other than to those of other groups. The model analyzes both fixed effects caused by the population, as well as random effects introduced by subject-specific effects and can therefore describe the presented data more accurately than calculating only the mean (19). These analyses were conducted in R (Version 4.1.2, The R Foundation for Statistical Computing, Wien, AT), using the *lmer* function of the *lme4* package lme4 (Version 1.1–34) and the *confint* function of the *stats* package (Version 4.1.2). The linear mixed-effects model was set up with the mean N.I.H. per experiment and pump as the dependent variable, the pump type as the independent variable, and the experimental sequence as a random intercept-effect (Equation 2).

(2) $$\begin{array}{c} N.I.H.\;∼\;Pump\;Type\;+\;(1|Experiment)\; \end{array}$$

Each data point was weighed with the number of samples used to calculate the corresponding mean. The confidence intervals were calculated one-sided with the difference between the prototypes minus the corresponding reference pump. Therefore, positive values indicate an increased hemolysis index for the prototype compared to the respective reference pump. The lower bound was fixed at minus infinity since the prototypes are not expected to produce a substantial lower value of hemolysis in comparison to the reference pump.

The maximum error in the N.I.H. accounting for measurement errors of fHb and Hct was calculated by generalizing these errors to a difference in fHb of ±1 mg/dL. Calculating the N.I.H. with this assumption (at a hematocrit of 32%), a difference in N.I.H. of less than 9.06 mg/100 L would indicate that the hemolysis of the prototype is similar to that of the reference pump.

## 3 Results

**Figure 3:**
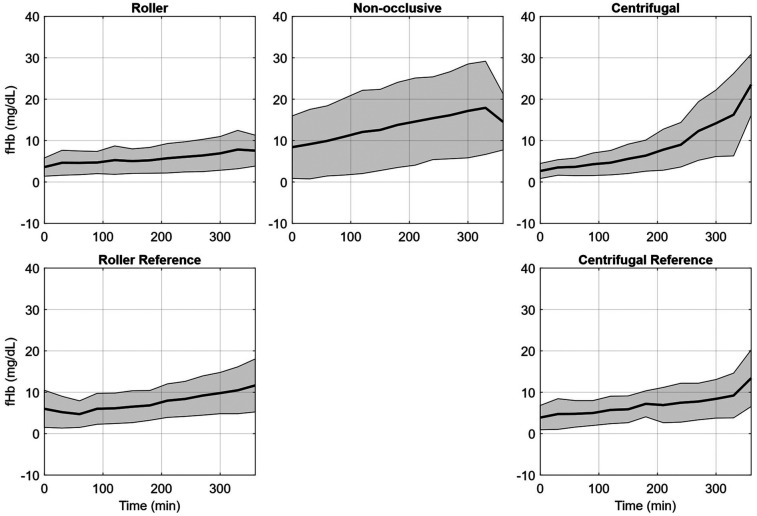
Free plasma hemoglobin (fHb) averaged over the seven hemolysis experiments for the roller pump (RP), non-occlusive roller pump (NRP), centrifugal pump (CP), reference roller pump (RP Ref), and reference centrifugal pump (CP Ref). The plots indicate the mean fHb values (black line) and the respective standard deviation (grey area).

In Table 1, the N.I.H. is reported separately for each repetition of the experiment as an mean with one standard deviation (SD). For the roller pump 80 (95%) of the data points could be included, for the non-occlusive roller pump 80 (95%), for the reference roller pump 67 (80%), for the centrifugal pump 62 (74%), and for the reference centrifugal pump 64 (76%). Data were excluded if (1) water entered the circuit and caused severe hemolysis (fHb rose much faster than normal and there was visual discoloration of the blood), as in Experiment 2 for the centrifugal reference pump after 2.5 h, (2) visual problems occurred with the sample taken, such as discoloration of the plasma after centrifugation (Experiment 1, datapoint ‘0 h’, roller pump), (3) premature stop of a circuit due to leaks (Experiment 1, after 3 h, Centrifugal Pump) or (4) mechanical failure of the circuit (Experiment 2, roller reference, tubing breaking inside the pump head due to user error). Further details on the missing individual data points are listed in Supplementary S1. To provide a deeper insight into the change of the fHb value over the duration of the experiment, Figure 3 shows the mean increase of fHb value during the experiments for each of the pumps, Table 2 shows the mean fHb values at the start (0 h) and end (6 h) of the experiment, as well as the mean slope.

**Table 1:** Results of the calculated mean normalized index of hemolysis (N.I.H.) with the corresponding standard deviation (SD) per experiment for all three developed pumps, as well as the two reference (Ref) pumps. The number of included data points is given in brackets for each experiment and pump (n).

Normalized index of hemolysis [mean ± SD (n)] (mg/100 L)					
Experiment	Roller	Non-occlusive	Roller Ref	Centrifugal	Centrifugal Ref
1	1.52 ± 9.00 (11)	11.78 ± 11.23 (11)	6.54 ± 11.67 (10)	2.42 ± 5.48 (6)	1.97 ± 12.64 (10)
2	1.10 ± 3.12 (9)	8.72 ± 4.14 (9)	4.67 (1)	2.41 ± 2.15 (11)	3.17 ± 5.60 (4)
3	2.13 ± 2.61 (12)	6.62 ± 4.62 (12)	2.42 ± 2.35 (10)	4.77 ± 3.98 (7)	1.09 ± 3.37 (6)
4	2.22 ± 1.50 (12)	4.91 ± 6.90 (12)	7.82 ± 8.81 (10)	18.79 ± 11.72 (12)	10.16 ± 6.45 (12)
5	3.15 ± 11.00 (12)	7.26 ± 11.79 (12)	10.32 ± 7.41 (12)	16.41 ± 7.40 (12)	6.95 ± 4.30 (12)
6	1.28 ± 2.24 (12)	9.62 ± 4.54 (12)	1.70 ± 1.62 (12)	3.80 ± 2.71 (8)	1.98 ± 1.00 (8)
7	1.53 ± 2.39 (12)	4.72 ± 2.11 (12)	1.94 ± 3.16 (12)	2.50 ± 3.57 (6)	1.99 ± 2.80 (12)

**Table 2:** Detailed fHb values (mean ± standard deviation) at the start (0 h) and end (6 h) of the experiment, as well as the calculated increase per hour for the roller pump (RP), non-occlusive roller pump (NRP), centrifugal pump (CP), reference roller pump (RP Ref), and reference centrifugal pump (CP Ref).

Pump	fHb @ 0 h	fHb @ 6 h	fHb/h
RP	5.23 ± 4.77 mg/dL	8.21 ± 3.80 mg/dL	0.50 ± 1.02 mg/dL/h
NRP	8.41 ± 7.55 mg/dL	20.13 ± 11.67 mg/dL	1.95 ± 2.32 mg/dL/h
RPRef	6.00 ± 4.48 mg/dL	15.75 ± 8.77 mg/dL	1.63 ± 1.64 mg/dL/h
CP	2.66 ± 1.81 mg/dL	18.47 ± 10.17 mg/dL	2.64 ± 1.72 mg/dL/h
CPRef	3.89 ± 2.93 mg/dL	13.41 ± 6.84 mg/dL	1.59 ± 1.24 mg/dL/h

The mean measurements of pH are listed in Table 3. The measurements at the start of the experiments show a pH far below what is considered physiological. Over time the pH slowly increased for all pumps.

**Table 3:** Mean and standard deviation of pH measurements averaged over all seven experiments for the roller pump (RP), non-occlusive roller pump (NRP), centrifugal pump (CP), reference centrifugal pump (CPRef) and reference roller pump (RPRef).

pH (-)							
Pump	0 h	1 h	2 h	3 h	4 h	5 h	6 h
RP	6.81 ± 0.03	6.91 ± 0.04	6.99 ± 0.04	7.00 ± 0.12	7.00 ± 0.20	7.08 ± 0.18	7.65 ± 1.48
NRP	6.86 ± 0.04	7.04 ± 0.09	7.20 ± 0.15	7.20 ± 0.20	7.16 ± 0.27	7.17 ± 0.26	7.11 ± 0.20
RPRef	6.86 ± 0.04	6.99 ± 0.06	7.09 ± 0.07	7.18 ± 0.06	7.22 ± 0.03	7.18 ± 0.07	7.05 ± 0.24
CP	6.81 ± 0.04	6.86 ± 0.04	6.90 ± 0.04	6.91 ± 0.07	6.97 ± 0.11	7.01 ± 0.12	7.04 ± 0.08
CPRef	6.86 ± 0.08	6.93 ± 0.05	6.90 ± 0.04	6.96 ± 0.10	6.93 ± 0.15	7.00 ± 0.08	6.84 ± 0.23

MCV remained largely stable throughout each experiment, with nearly identical values across all five pumps within the same run (Table 4). Variations were observed primarily between experiments, reflecting differences in donor animals. In experiment 2, the reference centrifugal pump showed a sudden increase from 45 to 50 fL toward the end, coinciding with water contamination of the circuit and subsequent severe hemolysis. In experiment 3, the same pump exhibited a smaller increase from 50 to 53 fL. In experiment 6, a decrease from 47 to 44 fL at the end in the non-occlusive roller pump resulted in a higher standard deviation.

**Table 4:** Results of the calculated average mean corpuscular volume (MCV) with the corresponding standard deviation (SD) per experiment for all three developed pumps, as well as the two reference (Ref) pumps.

Mean corpuscular volume (mean ± SD) (fL)					
Experiment	Roller	Non-occlusive	Roller Ref	Centrifugal	Centrifugal Ref
1	43.71 ± 0.45	43.86 ± 0.35	44.00 ± 0.00	44.00 ± 0.00	43.86 ± 0.35
2	44.71 ± 0.45	45.00 ± 0.00	45.00 ± 0.00	44.86 ± 0.35	46.67 ± 2.05
3	48.86 ± 0.64	49.43 ± 0.73	49.00 ± 0.76	49.20 ± 0.75	50.60 ± 1.20
4	48.14 ± 0.35	48.14 ± 0.35	47.86 ± 0.35	48.00 ± 0.00	48.00 ± 0.00
5	45.86 ± 0.35	45.71 ± 0.45	45.86 ± 0.35	46.14 ± 0.35	45.86 ± 0.35
6	45.71 ± 0.70	46.43 ± 1.05	45.71 ± 0.70	46.20 ± 0.40	46.40 ± 0.49
7	45.00 ± 0.00	45.57 ± 0.49	45.57 ± 0.49	45.25 ± 0.43	45.29 ± 0.45

**Figure 4:**
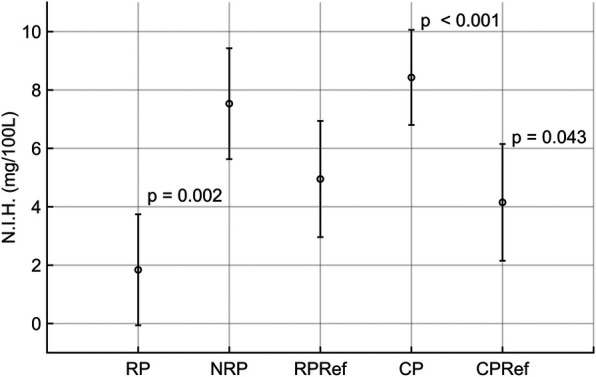
Visualization of the results of the linear mixed-effects model results, showing the calculated mean and standard deviation for the roller (RP), non-occlusive roller (NRP), centrifugal (CP), reference centrifugal (CPRef) and reference roller pump (RPRef). Results with statistical significance have the corresponding *p*-value reported at the top right of the respective barplot.

The results for the linear mixed-effects model are divided into fixed effects, random effects, and 95% confidence intervals for the N.I.H. (Table 5). The centrifugal pump was selected as the intercept, with the highest expected N.I.H. value of 8.43 mg/100 L. The roller pump had the lowest expected N.I.H. value of 1.84 mg/100 L. The calculated estimates and standard errors for each pump for the fixed effects are visualized in Figure 4.

**Table 5:** Estimated normalized index of hemolysis (N.I.H.) with standard error according to the results of the linear mixed-effects model.

Linear mixed-effects model results			
Fixed effects	Estimate	SE	*p*-value
Intercept (Centrifugal)	8.43	1.63	**<0.001**
Centrifugal ref	−4.28	2.00	**0.043**
Roller	−6.59	1.90	**0.002**
Non-occlusive	−0.90	1.90	0.640
Roller ref	−3.48	1.99	0.092
Random effects	Variance	SD	
Experiment (Pooled)	4.29	2.07	
95% confidence intervals	Estimate	Upper	
Roller Pump ≥0	−3.11	0.79	
Non-occlusive ≥0	2.58	6.48	
Centrifugal ≥0	4.28	8.48	

The table shows the results of the statistical evaluation of the hemolysis experiment. The evaluation was done with a linear mixed-effects model; the estimate, standard error (SE), and *p*-value of the fixed effects are given in the top part of the table, with the Centrifugal pump group as intercept, the random effects with variance and standard deviation (SD) in the middle part of the table and the 95% confidence interval estimates and their upper limits in the bottom part of the table. The lower limit of these intervals was omitted since the analysis was calculated only one-sided, resulting in lower bounds of minus infinity. Significant results are marked in bold. Note, the order is switched compared to other tables in this work to keep the intercept as the first entry.

The pooled random effects had a variance of 4.29 ± 2.07 for the N.I.H value. For the confidence intervals, the estimated difference between the roller pump and its reference was −3.11 with an upper boundary of 0.79 for the N.I.H value. The estimate for the non-occlusive roller pump was 2.58 with an upper boundary of 6.48 for the N.I.H value. For the centrifugal pump, the estimates were 4.28 with an upper boundary of 8.48.

## 4 Discussion

During open-heart surgery, an HLM supports the patient's cardiovascular function and oxygenation. This solution could also be extended to ECMO systems. In ECMO applications, such MR-conditional blood pumps would enable patients to undergo advanced imaging without interruption of life-sustaining therapy. To better understand brain perfusion dynamics, especially in pediatric patients, MRI offers a non-invasive method to evaluate brain tissue integrity and blood flow. MRI is commonly used to detect white matter injuries in infants before and after surgery (3–7). To facilitate MRI during HLM support, three MR-conditional pump prototypes were developed: a roller pump, a non-occlusive roller pump, and a centrifugal pump. Their hemolytic properties were evaluated using an *in vitro* blood circulation model to gain an initial impression of whether the prototypes could be used in an *in vivo* study. The pressure-head setpoint is well below a physiological value, as the centrifugal pump cannot generate a relevant pressure head. To ensure comparability between the pumps, the same setpoint was used for all three prototypes and the two corresponding reference pumps.

Standardized, processed bovine blood was used instead of abattoir-sourced blood to reduce inter-experimental variability (16, 20). The blood had an fHb value of 5.24 ± 4.86 mg/dL at the start of the experiment. Variability in initial fHb values between runs, despite being from the same animal, likely resulted from inconsistencies during circuit filling. Each experiment used four 500 mL bottles of blood, which were pooled into a single container to ensure uniformity across circuits. This required manual pouring into the reservoir rather than closed-system transfer from a blood bag, potentially contributing to the observed deviations in fHb values.

Several technical problems led to missing or unusable data. Mechanical leaks in the centrifugal pump necessitated increased priming volume to maintain a runtime of 6 h. Additionally, water entered the reservoir due to leaky connections, which led to further data loss. In total, 9 datapoints (2.1% of all data) were excluded due to sampling issues. 31 datapoints (7.4% of all data) were excluded due to water ingress in the circuits. 25 datapoints (6.0% of all data) were excluded due to premature stoppage of the circuits because of leakage or tubing rupture. Despite these challenges, 84% of the data could be used for analysis, enabling a preliminary comparison of the prototypes. Both reference pumps showed comparable increases in fHb values over six hours. While centrifugal pumps are generally associated with lower hemolysis than roller pumps, literature suggests that at low flow rates, centrifugal pumps may cause greater damage due to prolonged blood residence time within the pump head (21).

Among the prototypes, the roller pump demonstrated noticeably lower increase in fHb levels than its commercial reference pump, while the non-occlusive roller pump showed a higher increase. The centrifugal pump prototype exhibited greater hemolysis than its reference pump, even though both used the same pump head design. The generally low hemolysis values for all configurations can be attributed to the study's low pressure head and flow conditions.

The measured pH values were initially below physiological levels, consistent with the use of acid-citrate dextrose in blood preparation. Over the course of the experiments, the pH value rose gradually but generally remained below the typical physiological value of ∼7.3. The exact cause of this increase could not be clearly determined; however, literature suggests an inverse relationship between pH and pCO_2_, whereby an increase in pCO_2_ lowers the pH value (22). In our case, the observed increase in pH could indicate a decrease in pCO_2_, potentially due to diffusion into ambient air with lower CO_2_ partial pressure.

Overall, MCV values were stable across pumps within individual experiments, indicating that the setups themselves did not introduce systematic variability. Differences between experiments are most likely attributable to donor-related variability in hematologic parameters. The transient increases in MCV observed in the reference centrifugal pump during experiments 2 and 3 may be related to hemolysis, with the marked rise in experiment 2 plausibly caused by water entering the circuit and contaminating the blood. This increase is especially pronounced as the circuit was not immediately stopped upon identifying discoloration of the blood. While the measured fHb values, which reached up to 3.6 g/L, were discarded for the calculations, further measurements of MCV revealed a sudden increase from 45 to 50 fL, coinciding with an increase in the osmotic pressure gradient, which in turn led to destruction of the red blood cells (23). In contrast, the decrease observed in experiment 6 may reflect natural variability or technical factors toward the end of the run. Taken together, these findings suggest that, apart from isolated events, MCV remained largely unaffected by the experimental conditions.

The linear mixed effects model shows that the centrifugal pump had a higher estimated hemolysis than the roller pump potentially due to the low flow setpoint (21). However, when comparing the reference roller and centrifugal pumps, the centrifugal pump showed a slightly lower expected hemolysis index than the roller pump. The non-occlusive roller pump produced much higher hemolysis than expected. This might have been caused by using a different tubing than that used by Montoya et al. (24).

The confidence intervals show that the difference in the hemolysis indices between the prototypes and the corresponding reference pumps are below the threshold of 9.06 mg/100 L calculated from an fHb measurement error of ±1 mg/dL. This indicates that all pumps tested provoked similar hemolysis, with the roller pump producing the lowest value of fHb. Typically, prototype heads show higher hemolysis than commercial counterparts; however, the roller and centrifugal prototypes were modified commercial designs. Additionally, the occlusion for the reference roller pump may not have been set optimally for the experiments, but literature suggests that this setting has no influence on the overall hemolysis of the pump (25).

To account for variability between experimental runs, the model incorporated a random intercept for the grouping variable “*Experiment”*. This approach allows the model to estimate a unique baseline (intercept) for each experimental iteration, thereby accounting for unobserved heterogeneity that could influence the response variable “*N.I.H.”* independently of the pump type used. The estimated variance of the random intercept for *Experiment* was 4.29, corresponding to a standard deviation of 2.07. This indicates a moderate degree of between-experiment variability in baseline N.I.H. values.

In other words, after taking the pump type into account, different experimental runs still showed meaningful differences in their mean N.I.H. values. This variability could stem from multiple uncontrolled or unmeasured factors inherent in each experiment — such as slight differences in setup, operator technique, environmental conditions, equipment calibration, or batch effects, confirming the importance of using a linear model with mixed-effects over a simple calculation of mean values.

Most published studies on CPB blood pumps evaluate performance under physiological pressure ranges, which our centrifugal pump was unable to reach. Consequently, direct comparison of our N.I.H. values with most of the existing literature is limited. Although power-law models such as that of Giersiepen et al. (26), could in principle be used to extrapolate hemolysis at higher setpoints, differences in pump principles and geometries make these estimates inaccurate.

Some studies have reported hemolysis under pressure heads comparable to our experimental setpoint, albeit in applications other than CPB. Nevertheless, because hemolysis is strongly dependent on the pressure head, these findings provide a useful point of reference. Liu et al. (27) evaluated an intravascular micro-axial blood pump using porcine blood at flows of ∼1.25 L/min against a pressure rise of 10–15 mmHg, reporting N.I.H. values of 24.8 ± 13.2 mg/100 L. Giridharan et al. (28) investigated a pediatric viscous-impeller pump for cavopulmonary assist, operating with fresh bovine blood at 2.2 ± 0.3 L/min and 15 ± 2 mmHg, and reported N.I.H. values of 70 mg/100 L over six hours. Escher et al. (29) studied an impeller-based cavopulmonary assist device for Fontan patients, using bovine blood at 4 L/min and 12.55 mmHg, and observed N.I.H. values of 3.8 ± 1.6 mg/100 L.

In comparison, our prototypes tested at a pressure head of 10 mmHg exhibited N.I.H. values of 8.43 ± 1.63 mg/100 L for the centrifugal pump, 1.84 ± 1.90 mg/100 L for the roller pump, and 7.53 ± 1.90 mg/100 L for the non-occlusive roller pump. The corresponding reference pumps yielded 4.15 ± 2.00 mg/100 L for the centrifugal reference and 4.95 ± 1.99 mg/100 L for the roller reference. These findings indicate that hemolysis in our devices is of the same order of magnitude as other low-pressure systems reported, though pump-specific differences are apparent. Importantly, differences in flow setpoints, circuit design, and blood donor species across studies limit direct comparability and prevent extrapolation of our results to clinical practice.

The study experienced many limitations, reducing the importance of the results collected. One limitation reduced interpretability due to the choice of blood. Although bovine blood is often used in hemolysis studies, bovine red blood cells possess higher resilience to mechanical stress than human red blood cells and therefore, during our investigation blood cell damage is rather underestimated (30). The use of acid-citrate dextrose for anticoagulation caused the blood pH to reduce below physiological levels. The biggest limitation of this study is the low pressure head during the experiments. The centrifugal pump circuit could not create a larger pressure head, even after almost clamping the downstream tube entirely and running the pump at full speed. The setpoint of 10 mmHg was then applied to all five circuits to ensure comparability of the results. This low pressure head is likely the result of both mechanical and experimental design-related factors of the centrifugal pump. Mechanically, insufficient pump speed (potentially caused by excessive friction in the actuation) and leakage in the centrifugal pump could have reduced output performance. From the experimental design standpoint, the use of open reservoirs could have prevented the system from building negative pressure on the pump inlet side due to the lack of resistance by the compliance of air inside a closed reservoir, further limiting the achievable head. Therefore, the results of the hemolysis study can only be used to compare the pumps but not to make a statement about the absolute hemolysis caused by the pumps. Additionally, due to replacing the magnetic coupling of the centrifugal pump with a mechanical coupling, the pump experienced significant leakage. Due to leaks in the reservoir, water from the water bath got into the tubing, which in turn led to the termination of this particular experiment for this pump. Finally, some samples were deemed unusable for analysis due to issues with sampling or preparation, which could have been prevented by taking two samples as the ASTM guideline proposes. While the remaining 84% of datasets provided consistent results that allowed for meaningful comparison between prototypes, in future work two samples should be taken per circuit at each time step according to the ASTM guideline.

## 5 Conclusion

An MR-conditional blood pump enables the use of MRI to detect early changes in cerebral perfusion during cardiac surgery. Our study shows that our roller, the non-occlusive roller, and centrifugal pump all show suitable low hemolysis at this low pressure head of 10 mmHg, with the roller pump inducing the lowest value of hemolysis. The centrifugal pump showed the largest increase in hemolysis and suffered from severe leakage through the mechanical transmission. Therefore, under these constrained experimental conditions, the roller pump exhibited the most favorable hemolysis profile among the three prototypes. While these findings cannot be generalized to clinical practice, they provide important proof-of-concept that MR-conditional pumps can be safely operated within the MR environment without prohibitive hemolysis. The key take-away is that the roller pump offers a promising baseline for further development, whereas centrifugal and non-occlusive roller designs require targeted refinements to overcome leakage and optimize performance. These insights guide future design directions for MR-conditional pumps, both for intraoperative MRI research and for potential integration into ECMO systems. The next step is to repeat the study under physiological conditions in strict compliance with ASTM F1841 to enable direct comparison with other studies and to further assess clinical relevance.

## Data Availability

The raw data supporting the conclusions of this article will be made available by the authors, without undue reservation.
