# Supplementary material for: Preliminary in vitro hemolysis evaluation of MR-conditional blood pumps
Source: Front Med Technol. 2025 Oct 23;7:1671938. doi: 10.3389/fmedt.2025.1671938 (PMC12589963; doi:10.3389/fmedt.2025.1671938)
Supplement: Supplementary file 1 [file Datasheet1.pdf]

# *Preliminary In-vitro Hemolysis Evaluation of MR-conditional Blood Pumps – Supplement Material*

## 1 Supplement 1 – Excluded and Missing Datapoints

The following section presents the normalized index of hemolysis (N.I.H.) values for each experiment. Data points excluded from statistical analysis are highlighted in red, with accompanying explanations provided for each exclusion.

### 1.1 Experiment 1

| h | RP | NRP | CP | CP Ref | RP Ref |
|---|---|---|---|---|---|
| 0.5 | -43.32 | 18.84 | -1.22 | 26.17 | -8.91 |
| 1 | -10.20 | 14.73 | 1.19 | -15.14 | -30.69 |
| 1.5 | 1.30 | 20.81 | 7.44 | -5.33 | 15.37 |
| 2 | 17.61 | 21.81 | -5.52 | 12.84 | 3.57 |
| 2.5 | -12.98 | -1.83 | 9.18 | -14.88 | 11.38 |
| 3 | 6.53 | 19.05 | 3.45 | 9.45 | -8.58 |
| 3.5 | 9.08 | 13.27 | | 5.46 | 14.27 |
| 4 | 1.00 | -8.69 | | 2.83 | -1.50 |
| 4.5 | 3.99 | 24.60 | | -3.96 | 16.03 |
| 5 | -4.06 | -1.98 | | 2.26 | -2.92 |
| 5.5 | 8.66 | 9.07 | | 14.91 | 20.76 |
| 6 | -4.18 | 36.66 | | 63.83 | 21.34 |

The first data point of the roller pump (RP) was excluded due to an error in sample extraction. The initial free hemoglobin (fHb) value was excessively high, resulting in a strongly negative N.I.H.

For the non-occlusive roller pump (NRP), the final data point was excluded, as well as the last two data points for the reference centrifugal pump (CP Ref). In both cases, water had entered the circuit, causing the blood to turn completely black. NRP included one additional data point because discoloration occurred earlier in the CP Ref circuit.

All data points after 3 h for the centrifugal pump (CP) were excluded. The pump had to be stopped prematurely due to severe mechanical leakage, which caused the reservoir to run dry.

The two excluded data points in the reference roller pump (RP Ref) were also due to an issue with sample extraction. The last two data points of the RP Ref were retained despite a sharp increase in N.I.H. between 5 h and 5.5 h. Since there was little change between 5.5 h and 6 h, water ingress was not considered the cause of the spike.

## 1.2 Experiment 2

| h | RP | NRP | CP | CP Ref | RP Ref |
|---|---|---|---|---|---|
| 0.5 | 1.52 | 9.82 | 1.37 | 0.47 | 5.28 |
| 1 | 1.56 | 9.95 | -0.12 | -2.75 | 53.77 |
| 1.5 | 2.04 | 6.22 | 1.41 | 4.77 | -45.03 |
| 2 | 0.27 | 11.11 | -0.28 | 10.18 | |
| 2.5 | -1.15 | 9.45 | 0.99 | 32.28 | |
| 3 | 0.99 | 5.12 | 3.07 | 331.06 | |
| 3.5 | 2.32 | 16.42 | 4.99 | 838.13 | |
| 4 | 7.05 | 8.68 | 2.87 | 979.69 | |
| 4.5 | -4.71 | 1.69 | 4.81 | 955.16 | |
| 5 | 11.56 | 19.77 | 6.14 | | |
| 5.5 | 27.69 | 33.11 | 1.25 | | |
| 6 | 32.44 | 56.98 | 13.48 | | |

Missing data points in the RP, NRP, CP, and CP Ref circuits were primarily caused by water intrusion into the blood circuit, which led to discoloration of the blood. In the CP Ref, this discoloration began particularly early, though additional data points were recorded beyond this point for exploratory purposes.

In the RP Ref, an issue occurred with the sample at the 1-hour mark, resulting in an abnormally high followed by a very low N.I.H., both of which were excluded. After 1.5 hours, the tubing in the pump ruptured, forcing an early termination of that experiment. Such an event is very severe, however, in this case it is attributed to user error, as we are certain the reference pump does not cause pump rupture per se.

## 1.3 Experiment 3

| h | RP | NRP | CP | CP Ref | RP Ref |
|---|---|---|---|---|---|
| 0.5 | 1.00 | -1.94 | 5.88 | -1.37 | 1.63 |
| 1 | 0.28 | 6.65 | -1.99 | 6.86 | 1.20 |
| 1.5 | 1.76 | 7.06 | 2.88 | -2.63 | 0.02 |
| 2 | -0.11 | 10.29 | 2.80 | 2.61 | 0.79 |
| 2.5 | -0.58 | 0.78 | 5.91 | -0.05 | 2.63 |
| 3 | 3.73 | 10.49 | 7.52 | 1.13 | 2.88 |
| 3.5 | 0.89 | 11.23 | 10.40 | | 6.85 |
| 4 | 4.70 | 10.08 | | | 1.75 |
| 4.5 | 1.58 | 0.83 | | | 0.20 |
| 5 | 0.86 | 4.83 | | | 6.18 |
| 5.5 | 8.85 | 11.91 | | | 59.83 |
| 6 | 2.63 | 7.19 | | | 116.40 |

The missing datapoints in the CP, CP Ref and RP Ref circuits occurred due to water entering the circuits.

## 1.4 Experiment 4

| h | RP | NRP | CP | CP Ref | RP Ref |
|---|---|---|---|---|---|
| 0.5 | 4.37 | -4.61 | 45.19 | 12.37 | 440.93 |
| 1 | 1.47 | 17.46 | 0.99 | 4.91 | -466.41 |
| 1.5 | 0.18 | -2.84 | 18.15 | 6.61 | 16.69 |
| 2 | 3.19 | 3.56 | 15.10 | 8.52 | -4.62 |
| 2.5 | 3.55 | 5.96 | 6.62 | 6.28 | -3.67 |
| 3 | 2.15 | 10.85 | 7.46 | 11.02 | 4.02 |
| 3.5 | 2.65 | -1.00 | 14.00 | 3.26 | 12.45 |
| 4 | 1.32 | 15.83 | 19.15 | 17.32 | 15.35 |
| 4.5 | 4.57 | 0.44 | 28.40 | -0.56 | 10.97 |
| 5 | 0.15 | 5.64 | 24.74 | 15.54 | 15.56 |
| 5.5 | 2.19 | 3.94 | 26.24 | 15.33 | -3.26 |
| 6 | 0.88 | 3.69 | 19.47 | 21.31 | 14.72 |

An issue with the sample caused the second fHb sample of the RP Ref to be unusable, in turn causing the first and second N.I.H. to be excluded from analysis.

## 1.5 Experiment 5

| h | RP | NRP | CP | CP Ref | RP Ref |
|---|---|---|---|---|---|
| 0.5 | -3.34 | 17.51 | 16.03 | 10.92 | 4.01 |
| 1 | 8.61 | -5.99 | 14.46 | 6.89 | 0.98 |
| 1.5 | -5.59 | 22.59 | 17.83 | 10.36 | 6.04 |
| 2 | 11.65 | 14.52 | 16.31 | 6.44 | 9.15 |
| 2.5 | -3.61 | 4.40 | 30.82 | 6.74 | 5.23 |
| 3 | -1.26 | 16.53 | 17.28 | -1.98 | 15.20 |
| 3.5 | 12.35 | -0.43 | 12.94 | 15.71 | 22.90 |
| 4 | 6.35 | 12.31 | 11.95 | 4.92 | 3.31 |
| 4.5 | 9.44 | 1.02 | 24.49 | 7.88 | 6.18 |
| 5 | 5.86 | 21.65 | 12.39 | 3.64 | 11.03 |
| 5.5 | 19.42 | -3.93 | 21.70 | 5.06 | 19.43 |
| 6 | -22.08 | -12.99 | 0.71 | 6.77 | 20.32 |

No data was excluded.

## 1.6 Experiment 6

| h | RP | NRP | CP | CP Ref | RP Ref |
|---|---|---|---|---|---|
| 0.5 | 2.15 | 2.94 | 7.25 | 0.73 | 0.99 |
| 1 | -1.04 | 3.24 | -1.35 | 1.32 | 0.83 |
| 1.5 | 2.32 | 11.43 | 3.95 | 1.64 | 2.31 |
| 2 | -0.86 | 5.28 | 1.77 | 2.37 | -1.66 |
| 2.5 | 0.22 | 6.55 | 3.94 | -0.81 | 1.15 |
| 3 | 1.73 | 10.79 | 6.70 | | 1.45 |
| 3.5 | 0.83 | 7.54 | 3.74 | 17.32 | 3.12 |
| 4 | -1.69 | 9.87 | 4.41 | 1.68 | 2.04 |
| 4.5 | 4.47 | 14.84 | | 3.68 | 4.18 |
| 5 | -0.57 | 15.28 | | 3.13 | -0.03 |
| 5.5 | 2.15 | 16.04 | | 1.32 | 2.36 |
| 6 | 5.63 | 11.64 | | | 3.69 |

The CP exhibited progressive mechanical leakage, necessitating termination of the run after 4 hours.

Two missing data points from the CP Ref resulted from sampling errors that rendered the blood unusable for analysis. An additional fHb value, though valid, was excluded to maintain consistency in the time intervals used for N.I.H. calculation.

## 1.7 Experiment 7

| h | RP | NRP | CP | CP Ref | RP Ref |
|---|---|---|---|---|---|
| 0.5 | 1.44 | 5.25 | 7.77 | 5.83 | -3.14 |
| 1 | -1.21 | 3.73 | 0.13 | -1.05 | 7.26 |
| 1.5 | 2.06 | 1.30 | -2.38 | -2.32 | -1.59 |
| 2 | 3.56 | 3.09 | 4.15 | 3.52 | -1.32 |
| 2.5 | 0.37 | 6.14 | 4.05 | 0.45 | 3.52 |
| 3 | -3.63 | 5.34 | 1.27 | 1.92 | 2.11 |
| 3.5 | 2.70 | 3.19 | | 2.29 | 1.41 |
| 4 | 1.21 | 4.57 | | -0.88 | 0.99 |
| 4.5 | 0.48 | 6.35 | | 5.31 | 6.55 |
| 5 | 4.81 | 9.66 | | 4.97 | 1.89 |
| 5.5 | 4.58 | 3.64 | | -0.40 | 1.04 |
| 6 | 1.93 | 4.38 | | 4.22 | 4.54 |

Leakage in the CP circuit progressively worsened, reducing the priming volume from 700 mL to just 150 mL within 3 hours. This significant loss necessitated an early termination of the run.
